# Grasping in One-Handed Catching in Relation to Performance

**DOI:** 10.1371/journal.pone.0158606

**Published:** 2016-07-08

**Authors:** Benedetta Cesqui, Marta Russo, Francesco Lacquaniti, Andrea d’Avella

**Affiliations:** 1 Centre of Space Bio-medicine, University of Rome Tor Vergata, 00133 Rome, Italy; 2 Laboratory of Neuromotor Physiology, IRCCS Santa Lucia Foundation, 00179 Rome, Italy; 3 Department of Systems Medicine, University of Rome, Tor Vergata, 00133 Rome, Italy; 4 Department of Biomedical and Dental Sciences and Morphofunctional Imaging, University of Messina, Messina, Italy; Purdue University, UNITED STATES

## Abstract

Catching a flying ball involves bringing the hand to the aimed interception point at the right time, adjusting the hand posture to receive the incoming ball and to absorb the ball momentum, and closing the hand to ensure a stable grip. A small error in any of these actions can lead to a failure in catching the ball. Here we sought to gather new insights on what aspects of the catching movements affect the interceptive performance most. In particular, we wondered whether the errors occurred in bringing the hand to the interception point or in closing the fingers on the ball, and whether these two phases of interception differed between individuals. To this end, we characterized grasping and wrist movement kinematics of eleven participants attempting to catch a ball projected in space with different ball arrival heights and flight durations. The spatial position of the ball and of several markers placed on the participant’s arm were recorded by a motion capture system, the hand joint angles were recorded with an instrumented glove, and several movement features were extracted. All participants were able to intercept the ball trajectory (i.e. to touch the ball) in over 90% of cases, but they differed in the ability to grasp the ball (success rate varied between 2% and 85%). Similar temporal features were observed across individuals when they caught the ball. In particular, all participants adapted their wrist movements under varying temporal and arrival height constraints, they aligned the time of peak hand closing velocity to the time of hand-ball contact, and they maintained the same hand closing duration in the different experimental conditions. These movement features characterized successful trials, and hence allowed to evaluate the possible sources of errors underlying unsuccessful trials. Thus, inter-individual and inter-trial variability in the modulation of each kinematic feature were related to catching performance. We observed that different participants used different solutions to bring the hand to the interception point. In particular the value of the wrist velocity at impact distinguished good from poor catchers. However, each individual showed similar wrist kinematics in grasped and touched trials. We also found that specific grasping features predicted the catching outcome, both on a trial-by-trial basis and across individuals of different performance level. A higher speed of hand closing distinguished touched from grasped trials. A proper triggering of the enclosing phase of the grasping movement and an accurate alignment of the peak of the hand closing speed to the impact event predicted the catching performance of different participants. These results indicate that the control of the grasping movement was the main source of errors affecting catching performance in our experiments. Moreover, these results suggest that distinct temporal and spatial features in the coordination of the grasping movement are related to individual catching abilities.

## Introduction

Interceptive actions such as catching require sophisticated spatio-temporal coordination of the arm and hand motion with target motion. The hand must reach the interception point at an appropriate time and must be oriented in a configuration suitable for grasping, so that the fingers firmly close around the ball [1, 2]. During hand transport, the fingers open to maximize the probability of receiving the incoming ball [1–5]. The grasping movement begins when the ball is still on air, and terminates after the ball contacts the hand. At the end, the hand’s grip on the ball is tightened to avoid its slippage [1]. Much research on one-handed catching has focused on the characterization of arm and grasping kinematics in search of basic control policies with which people coordinate their motor response in relation to the ball motion. To this end, the analyses carried in the majority of studies included only successful trials. They emphasized the stereotyped nature of the arm transport component, which is coupled with ball motion and scales in amplitude and time as a function of ball speed [2, 4, 6–10]. Moreover, they reported that hand closing starts at a constant time before impact regardless of ball speed and experimental conditions [1–5, 9, 11]. However, to date, a systematic analysis of the possible sources of errors underlying unsuccessful catching is lacking. Because of the spatial and temporal constraints involved, the smallest error in any component of the interceptive action could lead to an unsuccessful catch [12, 13]. What are the interception components that affect catching performance most? For instance, errors may occur in the hand transport such that the hand does not intersect the ball trajectory or it intersects the trajectory at the wrong time. Alternatively, the hand may intersect the trajectory at the right time but the fingers may close too early or too late. Another issue is whether and how movement errors differ among individuals. Several studies on interceptive actions have reported that, if the movements are not constrained to a predefined path and participants are free to choose the interception point along the ball trajectory, almost infinite motor solutions can accomplish the task [1, 6, 14–18]. Therefore, it is reasonable to hypothesize inter-individual variability also in movement errors depending on the movement strategy and skill level.

Many studies have suggested that inadequate motor responses arise due to inaccurate target motion perception or prediction by examining participants with different level of expertise in specific interceptive tasks [19–24]. A common approach is to compare anticipation in visual search behaviors, decision making, and accuracy in movement execution between elite and novice ball sport players. This approach is grounded on the idea that expert players, having a more accurate representation of the task than novices, are more likely to succeed in the task. Thus, the analysis of the differences between the motor behaviors of the two groups allows identifying the functional role of specific movement features underlying high success probability. For example, one of the marks of the professional cricket batsmen oculomotor behavior consists in performing saccadic eye movements at the bounce point as accurately as possible, because this point is crucial for the evaluation of the post-bounce trajectory [22, 23]. Similarly, it has been shown that elite baseball players pursue with the eyes the ball throughout its flight longer and more reliably than non-expert players [20, 25]. However, it remains unclear whether and how the different movement aspects, which distinguish on average elite from novice players in a specific task, are also directly responsible for the participant’s performance in individual trials. To address this issue, it is necessary to relate the outcome of the interceptive action (i.e. a binary response variable) and the variation of critical kinematic variables with a logistic regression [26]. In this context, we recently provided evidence that, in a one handed catching task, participants were able to touch the ball in more than 90% of the cases and to visually track the target reliably, but they differed broadly in their catching performance (from 2% up to 80% of grasped trials over the total number of ball launches) [27]. These results indicate a good accuracy in the control of hand transport. However, some participants failed to grasp the ball effectively for reasons that, at that time, we were unable to determine.

In the present study, we aimed to fill this gap. To this end, we asked twelve subjects to catch a flying ball projected in space with different spatial and temporal characteristics. Grasping and arm movement kinematics were measured using a sensorized glove and a motion capture system and they were characterized by means of several parameters. By comparing these parameters both in successful (grasped) and unsuccessful (touched or missed) trials and across participants with different catching performance, we sought to gain insights on what aspects of hand transport and grasping movement affected catching performance most. Therefore, the present analysis had three goals: 1) to investigate the catching outcome and movement characteristics in response to the different ball flight conditions; 2) to assess whether successful and unsuccessful trials could be discriminated on the basis of the values of specific kinematic parameters; 3) to evaluate whether the catching probability was predicted by the individual values of specific kinematic parameters and thus to assess whether and how individual motor behaviors could distinguish between good and poor catchers.

## Materials and Methods

### Subjects

Data from a group of 11 participants (7 males and 4 females), between 22 and 42 year old (S_1_: 22, S_2_: 30, S_3_: 28, S_4_: 26, S_5_: 29, S_6_: 32, S_7_: 28, S_8_: 26, S_9_: 42, S_10_: 33, S_11_: 27) were used for the present analysis. All participants performed a one-handed catching experiment. Two of them were the first and last author of the study (S_5_ and S_11_ respectively). All subjects had normal or corrected to normal vision, were informed about the procedure and the aims of the study, which was approved by the Ethical Review Board of the Santa Lucia Foundation, and gave their written informed consent to participate to the experiment.

### Task and apparatus

**Fig 1:**
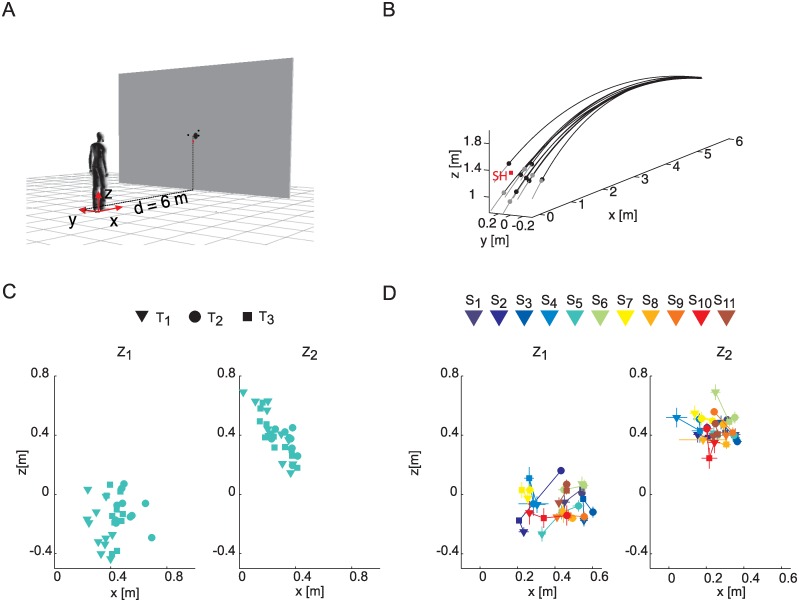
Experimental apparatus, representative examples of the ball trajectories and distribution of the impact points in the sagittal plane. (A) Overview of the experimental setup. Participants stood at a distance of 6 m from a large screen occluding from view a custom-made launching apparatus which projected balls in space with different flight durations and arrival heights. (B) Ball paths in the T_2_Z_1_ condition for subject S_5_; black lines represent the ball paths recorded by the motion capture system from the launch to the impact time; grey lines indicate the portion of the trajectory extrapolated from the impact position to the frontal plane passing through the participant's shoulder (SH); dots indicate impact positions along the ball trajectory in the case of successful (black dots) and unsuccessful (gray dots) trials. (C) scatter plot of the impact positions in the sagittal plane (x, anterior-posterior axis; z vertical axis) for subject S_5_ in all the trials; the different flight duration conditions are indicated by different marker shapes; data are shown separately for Z_1_ and Z_2_ conditions. (D) impact positions in the sagittal plane averaged across trials in each one of the three flight durations are illustrated for each participant and separately for two ball arrival heights. Participant color coding is shown on top of the panel.

Participants were asked to catch a ball projected by means of a dedicated launching apparatus [28], consisting of commercial launching machine (BOLA professional Cricket Bowling Machine, Stewart and Williams, Bristol, UK), mounted on a custom-made actuated structure, while standing at a distance d = 6 m. The launching system was located behind a large big screen to avoid visual anticipation (Fig 1A). The launching apparatus was calibrated [28] before starting the experimental sessions. The calibration procedure provided a mapping between the ball launch parameter (i.e. the speed at which the ball was released by the launcher and the orientation of the actuated structure) to be set on the display of the launching apparatus and the corresponding ball flight path characteristics. Six ball flight conditions, obtained by the combination of three mean ball flight durations (T_1_ = 0.55 s, T_2_ = 0.65 s, T_3_ = 0.75 s) and two mean ball arrival heights (low, Z_1_, and high, Z_2_), were tested similarly to our previous studies [6, 17, 27]. In the case of the subjects S_1_-S_10_, the Z_1_ and Z_2_ ball arrival heights were adjusted according to the shoulder height of the subject (H_sh_): Z_1_ = H_sh_—0.3 m, Z_2_ = H_sh_ + 0.3 m. In the case of participant S_11_ who performed the experiment at the very beginning of the experimental session, Z_1_ was 1.3 m and Z_2_ was 1.9 m (Z_1_ = H_sh_—0.22 m, Z_2_ = H_sh_ + 0.35 m).

Participants performed one block of 10 trials for each T-Z condition, for a total of 6 blocks. At the beginning of each block the settings on the launching machine were adjusted in order to project the ball in space with the initial ball velocity required to achieve the desired flight conditions. The order of the blocks was randomized across participants. Prior to the beginning of the experimental sessions participants familiarized with the task by performing a few catching trials. Each trial started with an auditory cue to alert the participant of a new launch. After the cue, the experimenter inserted the ball inside the launching machine.

Expanded polyurethane balls covered with a retro-reflective tape (Scotchlite, 3M, Pioltello, Milan. Italy) were used to track the ball during its flight. The spatial position of several retro-reflective markers placed on the participant’s body, and the position of the ball throughout its entire flight were tracked at 100 Hz using a motion capture system (Vicon-612, Vicon Motion Systems Ltd. UK). Markers were positioned on the skin overlying the right acromion, the epicondylus lateralis, and on the right forearm. The right wrist position (RW) was estimated by averaging the position of two additional markers placed at the extremity of a stick (length: 21 cm) taped on the participant wrist in correspondence to the mid-point between the ulnar styloid and radial styloid.

Marker coordinates were referred to a right handed calibration frame placed on the floor at 6 m distance from the launch plane (i.e. the world coordinate frame). Hand movements were recorded at 100 Hz using a glove instrumented with 22 piezoresistive joint angle sensors, i.e. the CyberGlove (CyberGlove Systems Inc., San Jose, CA). Finally, a mini-DV video camera (MID160, Canon, acquisition rate: 50 Hz) was used to film participants’ performance during the experiment.

### Data analysis

Each trial was classified as *grasped* if the ball was captured by the hand, *touched* if the ball was not captured but it contacted the hand, and *missed* if no contact occurred. Participant's catching performance was quantified as the percentage of successfully grasped balls over the total number of launches.

Grasping movements were characterized in terms of flexion/extension of the metacarpo-phalangeal joint of the index finger (MCP). For each participant data were normalized with respect to the maximum joint excursion (i.e. the difference between maximum and minimum values of the joint angle) measured throughout the session. Kinematic data from the Vicon markers and the CyberGlove were digitally low-passed filtered (25 Hz cutoff frequency, zero-lag FIR filter). Data were fitted with a cubic spline (Matlab csaps function), and differentiated to obtain the velocity and acceleration (Matlab, fnder function). Ball launch time was defined as the instant at which the ball passed through the screen. Impact time (IT) was computed as the instant at which the distance between the ball trajectory and the RW marker reached its minimum. The same procedure was applied to define IT also for missed trials even if no physical contact occurred.

**Fig 2:**
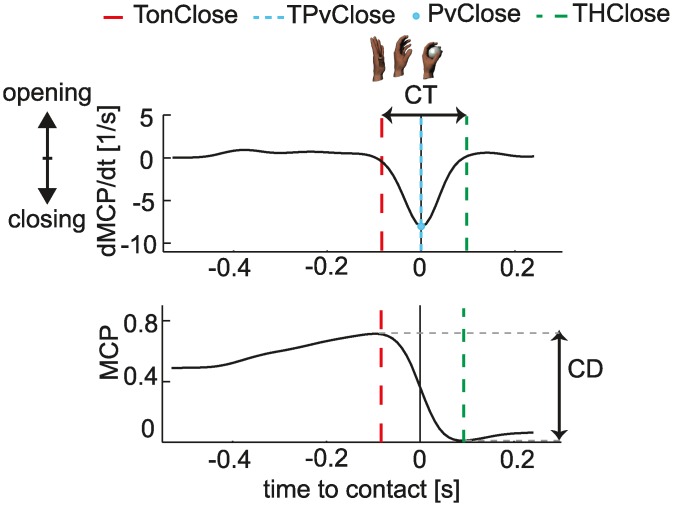
Main grasping features analyzed. Representative MCP angle in normalized units (bottom) and its time derivative (normalized angular velocity, top) profiles from subject S_9_ in the T_1_Z_1_ condition. Solid black lines indicate the time of the impact event. Vertical dashed red lines indicate the onset of the grasping movement (TOnClose). Cyan dashed lines represent the time of peak hand closing velocity. TPvClose is the duration of the time interval between the occurrence of the peak of closing velocity and the IT. Green dashed lines represent the end of the hand closing movement (THClose). The hand closing time (CT) is the duration of the time interval between TOnclose and THClose. Horizontal dashed lines indicate the maximum and minimum of MCP angle and define the closing excursion or distance (CD).

A few trials (41 over a total of 660) were not included in the analysis because the ball trajectory was not fully contained in the tracking volume of the Vicon system (about 7.5m × 3m × 3m) and could not be reconstructed throughout its flight. Our analysis focused on the hand closing movement which we hypothesized to be closely related to catching performance. The profiles of the MCP angle and its velocity in a sample trial (Fig 2) illustrate the main grasping features analyzed. The onset of hand closing or grasping movement (*TOnClose)* and the end of grasping movement (*THclose*) were defined as the duration of the interval from the time at which the MCP velocity dropped respectively below and above a threshold of 0.05 s^-1^ to IT. Closing Time (*CT*) was the time elapsed from *TOnClose*, onset of the grasping movement, to *THclose*, end of the grasping movement (i.e. *CT* = *THclose*—*TOnClose*). Total closing distance (*CD*) was defined as the difference between MCP maximal and minimal angles. Peak hand closing velocity (*PvClose*) was the largest negative velocity peak. Thus, peak hand closing speed was the absolute value of *PvClose*. The time of the peak hand closing velocity (*TPvClose*) was defined as the time at which *PvClose* occurred with respect to IT. Peak hand closing acceleration (*PaClose*) and its time (*TPaClose)* were defined as the largest negative acceleration peak from initiation of hand closure up to end of grasping movement and its occurrence with respect to IT.

Five kinematic and timing variables were computed to characterize hand transportation: 1) latency time (LT), i.e. the time elapsed from ball launch to wrist onset, defined as the time the tangential velocity exceeded a fixed threshold of 0.05 m/s; 2) wrist peak speed (WPs), i.e. the maximal tangential velocity evaluated from wrist onset to IT; 3) time to wrist peak speed (TWPs), i.e. the time elapsed from wrist onset to WPs occurrence; 4) the x (VIMPx) and 5) the z (VIMPz) components of the velocity vector at impact.

### Statistical analysis

We performed three analyses to investigate whether and how catching outcome (TEST 1) and kinematic parameters (TEST 2) depended on the ball flight characteristics and catching outcome depended on the individual average or the trial-by-trial variations of each kinematic parameter (TEST 3). A binary response variable Y was used to describe the catching outcome, i.e. Y_ij_ = 1 if the i-th participant grasped the ball at the j-th trial, Y_ij_ = 0 if the participants touched or missed the ball. We modeled such dependences by means of Generalized Linear Mixed Models (GLMM, [29, 30]) which express a qualitative dependent variable as a function of several independent variables in the manner of a linear (multiple) regression model. In particular, they extend traditional regression models by allowing the error term (the residual of the regression), or equivalently the response variable conditional to the independent variables, to be any distribution within the exponential family (a large family of probability distributions including the normal, Poisson, and binomial distributions) [31]. Moreover, GLMM allow assessing the dependence of the response variable both on fixed factors, i.e. the experimental variables (flight duration and ball arrival height) within subjects, and on a random factor between subjects. GLMM are used to estimate a single model across subjects, but they take into account that each subject could present a different variability and sample size, i.e. they consider that data can be organized in clusters depending on the subject [30, 32, 33]. In this respect, a crucial issue with GLMM is the identification of all the possible by-subjects dependencies in the sample (see S1 Appendix).

GLMM consist of three main elements: a random variable, represented by the dependent response variable R; a linear predictor which returns the mean of the response variable distribution, i.e. μ, and a link function (L) which is a function that transforms the expectation of the dependent variable E(R), i.e. the catching probability in the case of the Y variable, into a linear predictor as follows:

L [E(R)]= μ = β X​ (1)

where **β** is the vector of the fitted coefficients of the linear predictor, and **X** is the matrix of regressor variables. If R is a continuous variable, as in the case of kinematic parameters, the link function is the identity. If the response variable is binomial, as in the case of the binary Y variable introduced above, the link function is the probit function.

To determine the relation between catching probability and experimental conditions (TEST 1), a model of the type defined in eq 1, was fitted according to the following equation:

$$Y_{ij}{}^{*}=\beta_{0}+\beta_{T}t_{j}+\beta_{Z}z_{j}+S_{0i}^{}+\varepsilon_{ij}$$ (2)

where: i ϵ [1, …, number of subjects], and j ϵ [1, …, number of trials]; $Y_{ij}{}^{*}$ is a latent response variable [29]; *ε*_*ij*_ and *S*_0*i*_ are error terms which represent respectively the variability within (*ε*_*ij*_) and between subjects (*S*_0*i*_, see S1 Appendix); *t*_*j*_ and *z*_*j*_ indicate respectively flight duration and ball arrival height of the j-th trial. The p-values were evaluated according to the Wald statistics (*z*) [29].

Furthermore, we assessed whether there was a relation between the age of the participants and the catching performance (P) considering the following model: *P*_*i*_ = *β*_0_ + *β*_*A*_ *age*_*i*_ + *ε*_*i*_. For the sake of completeness an additional test was introduced to assess the dependence of the catching performance on the experimental conditions. To this end, the success rate (SR), i.e. the fraction of the successful trials over the total (i.e. the number of selected launches for each condition and participant), was also computed for each participant and each experimental condition. SR was then normalized according to arcsin(SR)^0.5^, and submitted to a two ways ANOVA with repeated measures (3 x time flight durations, 2 x arrival height as within-subjects factors). Significance and p-values were evaluated with F-test.

To assess the relation between several kinematic features and ball flight conditions, we considered a second test (TEST 2) involving GLMM. Kinematic parameters were related to the variables describing the T and Z experimental conditions (i.e. fixed effects) taking into account possible differences across participants (i.e. random effects). The analysis was performed only on grasped trials to assess the general trends and strategies adopted by the participants to accomplish the task. A detailed description of the procedure is provided elsewhere [27]. Briefly, for each kinematic feature analyzed, the model that fitted best the experimental data was identified first (see S1 Appendix for an introduction to random effects structure). To this end the *confirmatory hypothesis testing* approach was adopted [33], which consisted of building iteratively GLMM to test a set of specific critical hypothesis on the data structure [34]. The set of comparisons carried out are listed in Table 1 in sequential order. At each iteration, one simpler model and one more complex model were compared according to their likelihood ratio value (LR) [32, 33]. If the quality of the more complex model was lower than the quality of the simpler one the iteration ended, and the simpler model was selected. The estimation of the model coefficients and their significance was performed according to standard procedures, which use the likelihood ratio statistics (χ^2^) for the GLMM [32, 33]. In addition, a repeated measures ANOVA analysis (RM-ANOVA, 3 time flight durations x 2 arrival heights as within-subjects factors) was carried out on each movement feature evaluated to support the results of the GLMM (see S1 File and S1 Fig).

**Table 1:** Iterative approach to select the best model for each kinematic movement feature (ν) analyzed.

Iteration	Model	Specific critical hypothesis
1	*v*_ij_ = β_0_ + β_T_t_j_ + β_Z_z_j_ + S_0i_ + ε_ij_	GLMM with random intercept effect
2	*v*_ij_ = β_0_ + β_T_t_j_ + β_Z_z_j_ + γt_j_z_j_ + S_0i_ + ε_ij_	GLMM with random intercept effect and interaction between T and Z fixed effects. If the interaction term is significant is added to the later GLMM models
3	*v*_ij_ = β_0_ + (β_T_ + S_Ti_)t_j_ + β_Z_z_j_ + S_0i_ + ε_ij_	GLMM with random intercept effect and random slope with β_T_ effect
4	v_ij_ = β_0_ + β_T_t_j_ + (β_Z_ + S_zi_)z_j_ + S_0i_ + ε_ij_	GLMM with random intercept effects and random slope with β_z_ effect.
5	v_ij_ = β_0_ + (β_T_ + S_Ti_)t_j_ + (β_Z_ + S_zi_)z_j_ + S_0i_ + ε_ij_	GLMM with random intercept effects and random slopes with β_T_ and β_z_ effects.

Finally, to assess whether it was possible to predict catching probability from grasping and wrist kinematic features we performed a third analysis (TEST 3). Data from all participants and trials (i.e. grasped and non-grasped) in each T- Z experimental conditions were fitted according with GLMM defined by the following equation:

$$Y_{ij}{}^{*}=\beta_{0}+\beta (v_{ij}-{\bar{v}}_{i})+\delta_{i}({\bar{\nu }}_{i})+\varepsilon_{ij}+S_{0i}$$ (3)

where $Y_{ij}{}^{*}$ is the latent response variable as in Eq 2; *v*_*ij*_ is the kinematic parameter under consideration, ${\bar{v}}_{i}$ is its average value over the trials in the T- Z condition of the i-th participant. For each participant the $\left(v_{ij}{}_{}-{\bar{v}}_{i}\right)$ term (i.e. the *variation* term) evaluates the dependence of the response variable on the kinematic parameter once the mean value of the parameter for that participant ${\bar{v}}_{i}$ is taken into account. If *β* is significant, it means that there are differences in the *v*_*ij*_ value across successful and unsuccessful trials. The ${\bar{v}}_{i}$ term (i.e. the *within-subject average* term) is instead used to assess whether catching performance depends on the participant-specific mean value of the parameter. If *δ* is significant, it means that there are difference in the movement kinematics of individual subjects with different catching performances. The p-values were evaluated according to the Wald statistics (*z*) [29]. Statistical analyses were performed in the R software environment (R development Core team (2011). R foundation for statistical computing, Vienna. ISBN:3-900051-07-0 URL http://www.Rproject.org) with the lme4 package (lme4: Linear mixed-effects model using S4 classes. R package version 1.0–5 http://RAN.R-project.org/package=lme4). Multiple comparisons of means (i.e. Tukey Contrasts) were also performed with *multcomp* package.

## Results

The section is organized as follows. In the "Catching performance" subsection we present the results of the analysis on the relation between catching performance and ball flight characteristics, i.e. flight duration and arrival height. In the "General movement characteristics" subsection we describe how wrist and hand motions are modulated in response to ball flight characteristics in successful trials. A number of movement characteristics shared across participants in successful trials are then used as a reference for comparison with unsuccessful trials. Thus, in the "Catching performance in relation to individual average movement characteristics" subsection, the movements of participants of different skill level (i.e. different fraction of grasped trials over the total number of launches) are compared by relating the catching probability with the subject-specific average values of each movement parameter. This analysis identified critical wrist and grasping features distinguishing between good and poor catchers. Finally, in the "Catching performance in relation to movement characteristics on a trial-by-trial basis" subsection, the possible source of errors underlying catching failures in individual trials are assessed by relating the variation of specific movement features to catching probability.

### Catching performance

**Fig 3:**
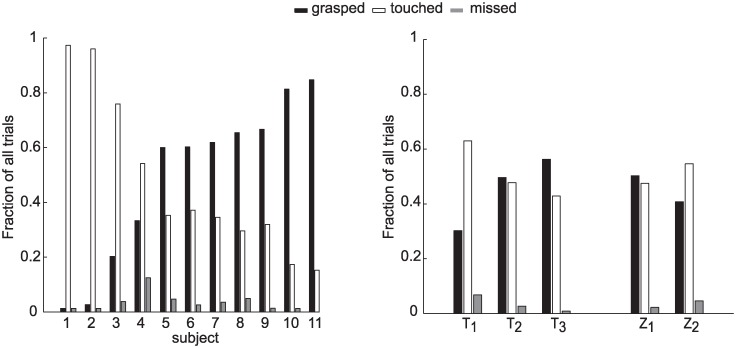
Percentage of grasped, touched, and missed trials throughout the experiment. Left panel: Participants are ordered according to their average catching success rate (i.e. the total number of the caught balls divided for total number of launches) from the lowest (i.e. S_1_) to the highest (S_11_) values. Right panel: fraction of grasped, touched and missed trials over the total (i.e. the number of the launches considering all the participants and the trials) for each T and Z experimental conditions.

The participants showed a broad distribution of catching performance (Fig 3) and they were ordered according to their average success rate (i.e. the total number of the caught balls divided by total number of launches), from the lowest, i.e. S_1_, 2% of successful trials, to the highest values, i.e. S_11_, 85%. The catching performance was not directly related to participant age (β_A_ = 2.03, p = 0.3). Catching success depended on ball flight characteristics. Statistical analysis (TEST 1, see Methods) indicated that there was a significant fixed effect of T, flight duration, on catching success (β_T_ = 9.50, p_T_ < 0.001). In particular, for all participants, catching success increased as T increased. No significant effect of Z, ball arrival height, was observed instead (p_z_ = 0.22). These results were confirmed by the RM-ANOVA test on SR, success rate. On average the performance changed depending on T (F_1,62_ = 32.46, p <0.001). However no significant differences were observed between the SR values in the two different ball arrival height conditions (F_1,62_ = 0.30, p = 0.58).

### General movement characteristics

We first assessed the relation between wrist and grasping kinematic parameters and T-Z experimental conditions. To this end only successful trials were considered (TEST 2). In our experiment no specific instructions were provided to the participants about where, when and how to intercept the ball. Fig 1 shows representative examples of the ball trajectories and the relative impact positions in one condition (T_2_Z_1_, Fig 1B), and in the other experimental conditions (Fig 1C) in the case of S_5_. Overall it provides evidence that the participants did not aim at directing their movement to specific locations (Fig 1D); they used visual information to guide their reaching behavior.

No significant effects of T, flight duration, and Z, arrival height, were observed for LT, wrist latency time (χ^2^_(1)_ = 3.02 p_T_ = 0.08, χ^2^_(1)_ = 0.06 p_Z_ = 0.8 Table 2). Fixed effects of T on the WPs, wrist peak speed, TWPs, time to wrist peak velocity, and VIMPz, vertical impact velocity, were significant (WPs: χ^2^_(1)_ = 73.31 p_T_ < 0.001, TWPs: χ^2^_(1)_ = 30.74 p_T_ < 0.001, VIMPz: χ^2^_(1)_ = 46.73, p_T_ < 0.001 Table 2). Participants moved faster, i.e. WPs and VIMPz increased, and TWPs decreased at higher ball approaching speeds, i.e. lower flight durations (WPs: β_T_ = -2.25, TWPs: β_T_ = 0.17, VIMPz: β_T_ = -3.41). Fixed effects of Z were significant for WPs, VIMP_x_, and VIMP_z_, but not for TWPs (WPs: χ^2^_(1)_ = 22.28 p_Z_ < 0.001, VIMP_x_; χ^2^_(1)_ = 83.12 p_Z_ < 0.001; VIMP_z_: χ^2^_(1)_ = 14.11 p_Z_ < 0.001, TWPs: χ^2^_(1)_ = 0.60 p_Z_ = 0.44). Participants moved faster, i.e. they showed higher WPs values, but they impacted the ball with a lower velocity along the posterior-anterior x-axis (Fig 1A) and with a higher vertical velocity component for higher balls arrival heights (WPs: β_z_ = 0.83, VIMP_x_: β_z_ = -0.53, VIMP_z_: β_z_ = 1.10). For all the wrist parameters fitted with GLMMs, the same direction of association between the motor response variables and the experimental conditions (i.e. the same sign of the regression coefficients) was observed once the by-participants adjustments of the T and Z slope coefficients were taken into account (i.e. β_T_ +S_Ti_, and β_Z_+ S_Zi_ coefficients, not reported for brevity). This means that, there was a similar modulation in the different individuals of the wrist motor responses as a function of temporal and spatial constraints induced by the ball motion.

**Table 2:** Results from the fitted ν responses variable for TEST 2.

Grasping parameters	T		Z		R^2^		Random slope T factor		Random slope Z factor	
	β_T_	p_T_	β_Z_	p_Z_	Marg.	Cond.	p_ST_	corr	p_SZ_	corr
Grasping										
TOnClose	0.08	0.14	-0.01	**	0.01	0.83	***	0.98		
CD	-0.37	***	-0.05	***	0.08	0.47				
TPvClose	-0.01	0.06	0.01	***	0.1	0.3				
PvClose	4.02	***	0.39	**	0.06	0.40				
TPaClose	-0.03	*	0.01	***	0.08	0.2				
PaClose	74.08	***	5.09	*	0.05	0.46				
CT	0.05	0.17	-0.01	0.40	0.00	0.68			**	-0.72
Wrist										
LT	0.13	0.08	-0.01	0.80	0.01	0.1				
WPs	-2.25	***	0.83	***	0.29	0.86			***	-0.92
TWPs	0.17	***	0.01	0.44	0.08	0.39			*	0.35
VIMP_x_	-0.15	0.67	-0.53	***	0.17	0.51				
VIMP_z_	-3.41	***	1.10	***	0.16	0.83			***	-0.98

The first column reports the name of the analyzed kinematic parameter. Subsequent columns report the regression coefficients (β) and p-values (p) of the fixed factors (flight duration T, ball arrival height Z). The fourth column reports the marginal (i.e. the proportion of variance explained by the fixed factor) and the conditional (i.e. the proportion of variance explained by both the fixed and random factors) R^2^ coefficient of the regression. Finally the two rightmost columns show the significance of the by-subject adjustment of the slope relatively to both the T factor (p_ST_), and the Z factor (p_SZ_), and the correlation between the two random effects (intercept and slope for each factor), when present. Numbers are rounded to the second decimal place. Results on the main effects are comparable to those obtained with the RM-ANOVA analysis reported in Table A in S1 File.

*: p_value <0.05;

**: p_value<0.01;

***: p_value<0.001.

**Fig 4:**
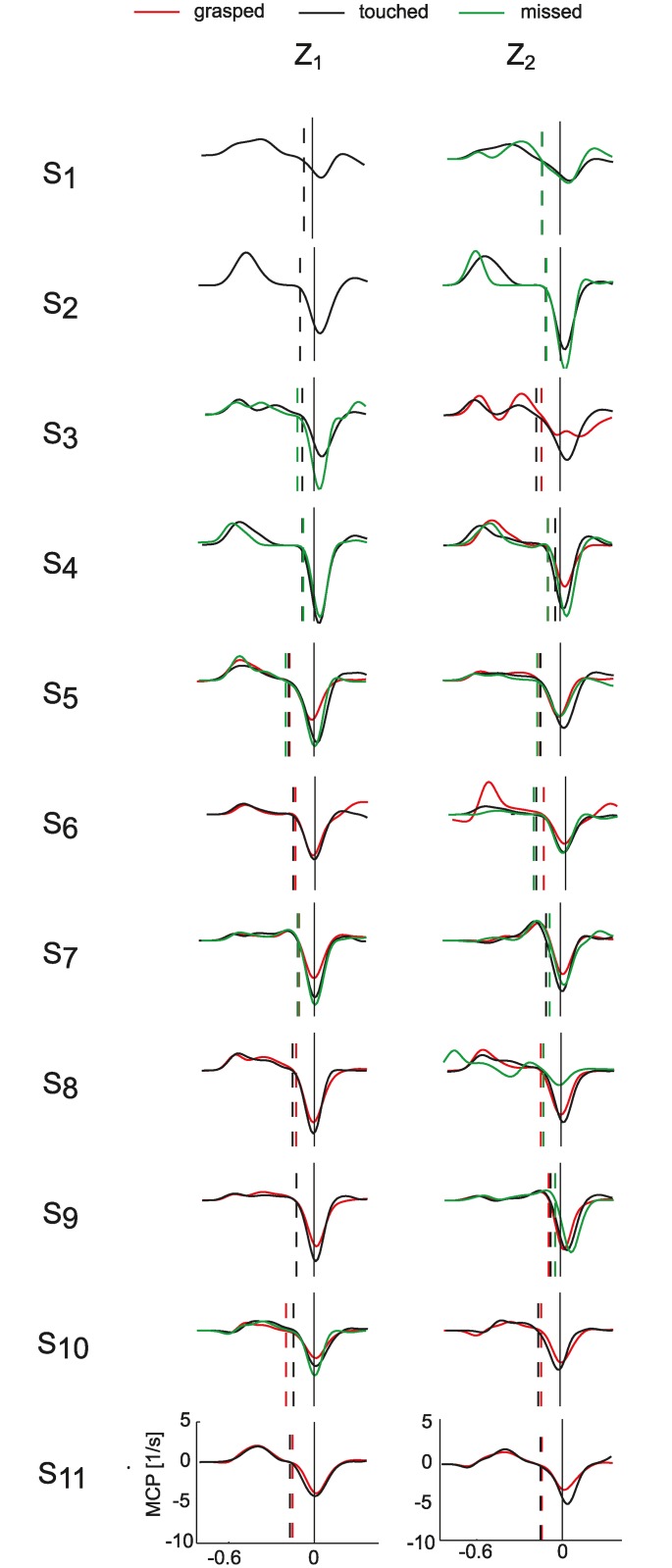
Grasping movement characteristics. Average MCP velocity profiles of grasped (red lines), touched (black lines), and missed (green lines) trials in the T_1_ conditions (Z_1_ left column; Z_2_ right column) are shown for all the participants (different rows). Data are aligned with respect to the impact event (black lines) and plotted up to 250 ms after the impact. Dotted lines represent TOnClose, time of hand closing initiation. Similar profiles were observed also in the other flight duration conditions T_2_ and T_3_. Participants are presented in an ascending order from S_1_ (top panel) to S_11_ (bottom panel).

All participants presented a similar time course of their grasping movement. Fig 4 shows the average normalized MCP joint velocity in the T_1_ conditions as a function of time to contact. In accordance with previous reports [4, 35], there were two distinct hand movement phases. In the first phase, the fingers opened and the angular velocity of the MCP joint presented positive values (as joint angle values increased with joint extension). In the second phase, the fingers started closing while the ball was still in the air and the MCP velocity was negative. The hand closure consisted in a simple ballistic flexion of the fingers around the ball, as shown by the bell-shaped velocity profiles of the MCP joint around the impact time (Fig 4). The fingers continued to close around the ball after impact to tighten the hand's grip. In accordance, the hand closing velocity returned to zero after the impact event.

**Fig 5:**
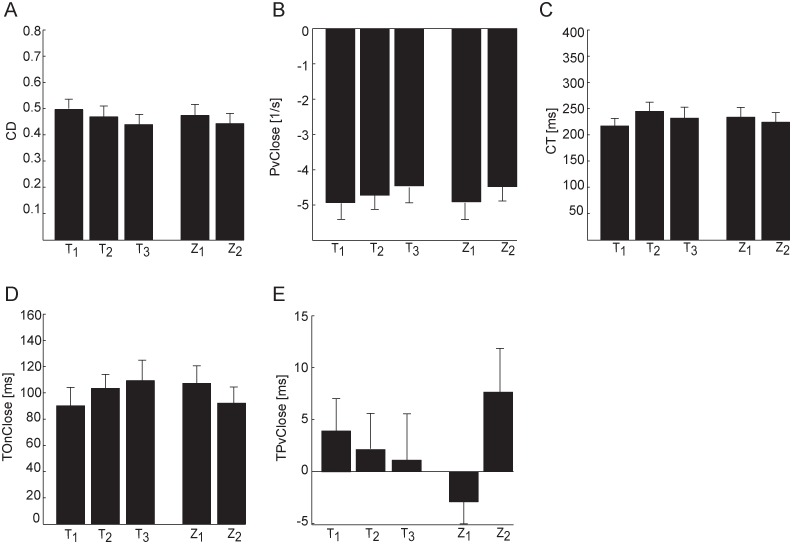
Average grasping parameters distributions across the different experimental conditions. Mean ± SE values (average across participants over grasped trials) are reported separately for each experimental condition. (A) CD, total flexion excursion. (B) PvCLose, peak of the MCP closing velocity. (C) CT, closing time. (D) TonClose, onset of finger closing movement. (E) TPvClose, time of the peak of the MCP closing velocity from impact.

In the case of higher balls, participants closed the hand less and at lower speed than for lower balls (Fig 5A and 5B). In accordance CD, closing distance, decreased and PvClose, peak closing velocity, increased (i.e. its absolute value, peak closing speed, decreased) as Z, ball arrival height, increased (CD: β_Z_ = -0.05, χ^2^_(1)_ = 16.44 p_Z_ < 0.001, PvClose: β_Z_ = 0.39, χ^2^_(1)_ = 8.08 p_Z_ < 0.01, Table 2). Moreover, participants decreased CD and moved slower (i.e. they decreased the absolute value of PvClose) for longer ball flight durations (CD: β_T_ = -0.35, χ^2^_(1)_ = 21.91 p_T_ < 0.001, PvClose: β_T_ = 4.02, χ^2^_(1)_ = 19.45 p_T_ < 0.001, Table 2). No significant relation between CT (Fig 5C), closing time, and T and Z were observed (χ^2^_(1)_ = 1.87 p_T_ = 0.17, χ^2^_(1)_ = 0.71 pz = 0.40).

TOnClose, onset of the grasping movement, increased with T (Fig 5D), passing from 91 ± 8 ms for T_1_, to 104 ± 10 ms for T_2_, to 110 ± 16 ms for T_3_ (mean ± SE across participants), but the effect of T was not significant (χ^2^_(1)_ = 2.12 p_T_ = 0.14). Significant effects of Z were observed instead (χ^2^_(1)_ = 9.15 p_Z_ < 0.01, β_Z_ = -0.01, Table 2). On average, participants began closing the index in the Z_2_, condition less than 10 ms earlier than in the Z_1_ condition, and specifically TonClose was 109 ±13 ms (mean ± SE across participants) in the Z_1_ conditions and 97 ± 13 ms in the Z_2_ conditions.

On average the TPvClose, the time of peak hand closing velocity with respect to the impact, passed from 4.9 ± 3.8 ms (mean ± SE across participants) in the T_1_ conditions to 2.1 ± 4.1 ms in the T_2_ conditions to 0.8 ± 5.4 ms in the T_3_ conditions (Fig 5E), although, according to TEST 2, the trend was not significant as TPvClose did not depend on T (χ^2^_(1)_ = 3.01 p_T_ = 0.08, Table 2). Participants increased the time of peak velocity values at higher ball arrival height conditions (χ^2^_(1)_ = 23.11, p_z_ <0.001, Table 2). In the Z_2_ condition (Fig 5E) TPvClose was on average 10 ms more than in the Z_1_ conditions (β_z_ = 0.01). In particular, TPvClose was -2.9 ± 3.6 ms (mean ± SE across participants) for Z_1_ and 7.6 ± 5.2 ms for Z_2_ (Fig 5D). However, such difference was small with respect to CT (230 ± 3.8 ms, mean ± SE across participants and conditions). Overall, in successful trials the hand-ball collision occurred approximately at the time of peak finger closing velocity rather than in correspondence of the raising (accelerated) or the descending (decelerated) portion of the finger closing velocity profile (Fig 4, red lines).

In summary, the analysis of successful trials identified a number of movement features shared by all participants. In fact they all presented the same relation (i.e. the same sign of the T and Z factors coefficients of the GLMM) between wrist movement parameters and ball flight characteristics, reached maximal hand closing speed approximately in correspondence of the impact time, increased the hand closing velocity and the hand closing distance for faster ball approaching speeds, and maintained the same hand closing duration in different experimental conditions.

### Catching performance in relation to individual average movement characteristics

In addition to the shared movement features in successful trials just described, we also observed marked differences across participants in several movement characteristics in accordance to previous studies [1, 6]. Since the participants enrolled in the study presented a broad distribution of the fraction of the grasped trials over the total (Fig 3), we investigated whether catching probability was predicted by the average values of the kinematic parameters of each individual. To this end, we fitted the data including both successful and unsuccessful trials with the GLMM reported in Eq 3 (TEST 3, see Methods). In particular, we evaluated whether individual catching movement characteristics were related to individual catching performance levels by assessing the significance of the within-subject average term in TEST 3 (the δ coefficient in Eq 3, see Methods). For each kinematic parameter, this term links catching probability to the average parameter value of each participant and hence it allows identifying those parameters that may contribute the most to the subject catching performance.

**Fig 6:**
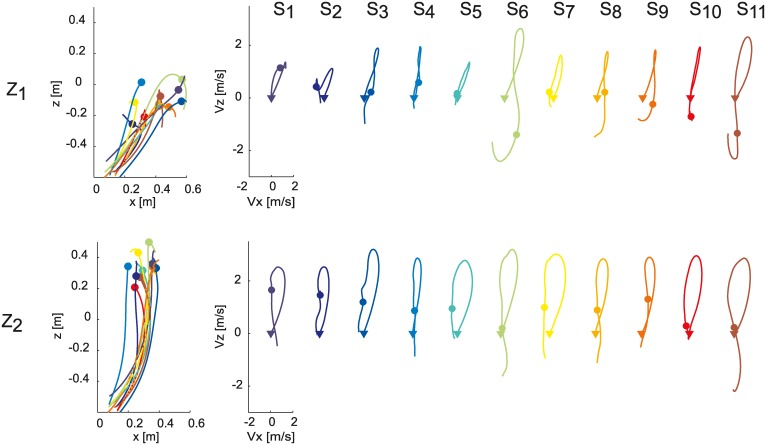
Differences in wrist kinematics across participants. Left column: average (mean across trials) wrist paths in the sagittal plane are shown for each participant in the T_2_ conditions (Z_1_ first row, Z_2_ second row). All trajectories are plotted up to 100 ms after the impact event (colored dots), and translated with respect to the shoulder position at launch time. Participant color coding is reported in the right panel. Right column: individual trajectories of the velocity components in the sagittal plane. Colored triangles indicate the velocity components at launch. Colored dots indicate the velocity components at impact.

As reported before [6], there were large differences across participants in wrist kinematics for one-handed catching in our experimental conditions. A detailed description of the different catching behaviors of a subset of the participants included in the present study, i.e. participants S_5_, S_6_, S_7_, S_8_, and S_11_, can be found in an earlier study [6], (respectively participants S_5_, S_2_, S_4_, S_3_, and S_1_ therein). Different participants impacted the ball at different locations along the ball trajectory (Fig 1, panel D). In low launches (Z_1_, Fig 6 top row), S_1_, S_2_, and S_4_ caught the ball away from the body, and presented high positive values of VIMP_z_, wrist vertical velocity at the time of impact (i.e. they used a *hitting-like* strategy). S_3_, S_5_, and S_7_ moved straight toward the target approaching the interception point from below and showed low VIMP_z_ values. We referred to this interceptive strategy as the *stop-on-the* ball strategy. Finally S_6_, S_8_, S_9_, S_10_, S_11_ presented a *hook-like* hand path, which consisted in an initial elevation of the wrist up to shoulder height and a following downward acceleration up to the final ball interception point. They hence arrived at the ball with a large negative VIMP_z_. In high launches (Z_2_, Fig 6 bottom row) inter-individual differences were less marked than in low launches. For instance, all participants caught the ball from below and arrived at the ball with a positive VIMP_z_, although less skilled participants (S_1_- S_4_) presented the highest VIMP_z_ values.

**Fig 7:**
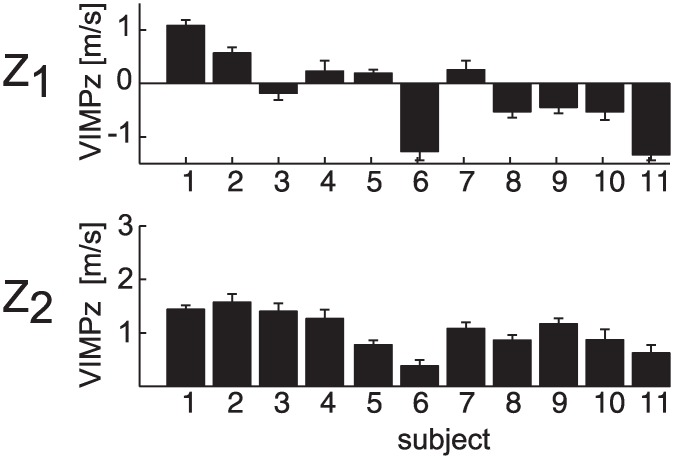
Wrist vertical impact velocity. The VIMP_z_ parameter (mean ± SE across trials in different T conditions) is reported separately for each participant and ball arrival height conditions (Z_1_: top; Z_2_: bottom).

Accordingly, the results of TEST 3 showed that poor and good catchers could be distinguished on the basis of the value of VIMP_z_, wrist vertical velocity at impact. In fact, the within-subject average term (i.e. the δ coefficient) was significant only in the case of VIMP_z_ (p_δ_ < 0.05, δ < 0, for all the T-Z conditions, with the exception of the T_2_Z_2_ condition, p_δ_ = 0.52, Table 3), indicating that catching probability was higher for those participants who presented a lower impact vertical velocity. Fig 7 shows VIMP_z_ values (mean ± SE across time flight conditions) for each participant separately in the two ball arrival height conditions. A lower (more negative in the case of Z_1_) VIMP_z_ predicted a higher catching performance. Conversely, participants with the worst catching performance (i.e. S_1_-S_4_) showed the highest (and mostly positive in the case of Z_1_) VIMP_z_.

**Table 3:** Results from the fitted Y* responses variable function for TEST 3.

	Variation-term (β)						Within subjects term (δ)					
	Z_1_			Z_2_			Z_1_			Z_2_		
	T_1_	T_2_	T_3_	T_1_	T_2_	T_3_	T_1_	T_2_	T_3_	T_1_	T_2_	T_3_
Grasping												
TOnClose												
pe	0.77	0.24	0.75	0.59	0.94	0.1	*	**	0.07	0.54	0.34	0.81
β, δ	-3.66	15.35	2.43	-4.55	0.58	8.76	53.05	41.51	47.79	9.7	30.81	-4.26
CD												
p-value	**	***	***	**	**	***	0.44	0.26	0.56	*	**	0.38
β, δ	-10.98	-11.16	-24.33	-6.41	-10.31	-15.24	-3.92	-7.34	-7.56	-7.46	-14.54	-7.94
TPvClose												
p-value	0.15	0.14	***	**	0.06	**	**	***	***	0.3	**	**
β, δ	-36.37	-27.59	-90.65	-43.47	-41.23	-87.37	-121.74	-122.61	-159.98	-46.72	-124.12	-97.77
PvClose												
p-value	***	***	***	***	**	***	0.52	0.52	0.79	0.06	**	0.79
β, δ	1.99	1.51	1.42	0.64	0.71	1.66	0.39	0.49	0.23	0.58	1.12	0.24
TPaClose												
p-value	0.13	0.27	**	*	0.11	**	**	***	***	0.79	**	**
β, δ	-38.61	-15.88	-80.6	-27.95	-32.97	-74.26	-130.66	-117.42	-172.32	-13.16	-126.34	-97.98
PaClose												
p-value	***	***	**	*	0.12	***	0.37	0.53	0.79	0.15	0.02	0.9
β, δ	0.07	0.07	0.06	0.02	0.02	0.05	0.02	0.02	-0.01	0.02	0.04	-0.01
CT												
p-value	0.92	**	0.75	0.83	*	0.23	0.66	0.55	0.29	0.66	0.72	0.2
β, δ	-0.57	25.63	1.97	1.32	6.67	8.99	9.52	11.81	20	5.81	6.91	-17.87
Wrist												
LT												
p-value	0.32	0.52	0.3	0.58	0.52	0.62	0.54	0.68	0.22	0.13	0.15	0.33
β, δ	5.33	3.1	-3.62	1.96	-3.75	-2.68	-14.23	10.18	17.52	27.02	29.87	25.15
WPs												
p-value	0.52	0.47	0.82	*	0.21	0.52	0.33	0.46	0.07	0.92	0.13	0.21
β, δ	0.35	0.59	-0.2	-1.33	-0.93	-0.57	0.96	0.9	4.36	0.17	4.42	1.85
TWPs												
p-value	0.37	0.48	0.69	0.39	0.53	0.84	0.29	0.32	0.26	0	0.41	0.4
β, δ	-6.8	3.82	-2.82	-3.73	-4.28	-1.74	-13.7	-14.62	-25.53	0.27	-15.37	8.71
VIMP_x_												
p-value	0.76	0.66	0.53	0.19	0.43	0.53	0.11	0.83	0.28	0.36	0.71	0.88
β, δ	-0.15	0.28	0.41	0.62	0.37	0.38	-1.54	-0.21	1.93	-1.03	-0.76	-0.22
VIMP_z_												
p-value	*	0.54	0.66	0.84	0.89	0.15	*	**	**	0.52	**	**
β, δ	0.78	0.26	0.22	0.07	-0.06	0.87	-1.47	-1.82	-2.81	-0.81	-3.3	-1.55

The first column report the name of the analyzed movement features. The subsequent columns report the regression coefficients and their p-values (p) of the variation terms (β), and the within subjects term (δ) for each T-Z experimental condition.

*: p_value <0.05;

**: p_value<0.01;

***: p_value<0.001.

Although the hand closing movement appeared more stereotyped than the wrist movement (Fig 4), differences between poor and good catchers were also present in the grasping kinematic parameters. Specifically, participants with the lowest catching performance (S_1_-S_4_) timed their hand closing movement less accurately. In particular, they showed delayed onset of the grasping movement (TOnClose) and time of peak closing velocity (TPvClose) with respect to IT, impact time.

The within-subject term in TEST 3 was significant for TOnClose in the T_1_Z_1_ (δ > 0, p_δ_ = 0.03) and T_2_Z_1_ (δ > 0, p_δ_ < 0.01) conditions (Table 3). Participants with the lowest catching performance (S_1_-S_4_) presented high TOnClose values in the fastest flight duration and lowest ball arrival height conditions. No significant relation between TOnClose and catching outcome was observed in the other conditions in all the participants.

**Fig 8:**
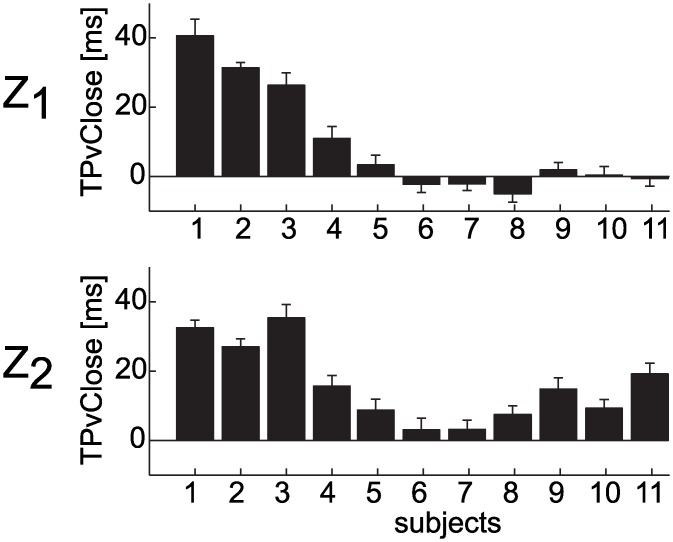
Timing of peak hand closing velocity. The distribution (mean ± SE across trials in different T conditions) of the TPvClose parameter is reported separately for each participant and Z conditions (Z1: top panel; Z2: bottom panel).

For both TPvClose and TPaClose, the within-subject average term was significant in all conditions (p_δ_ < 0.01, Table 3) with the exception of the T_1_Z_2_ condition (TPvClose, p_δ_ = 0.30, TPaClose, p_δ_ = 0.79, Table 3). Catching probability increased in relation to a decrease of the TPvClose average parameter (δ < 0 in all conditions). In accordance, the poorest catchers (S_1_-S_4_) showed higher values of TPvClose and lower values of TPaClose compared to the best catchers (S_5_-S_12_, Fig 8). The poorest catchers were not able to time hand closing accurately and TPvClose occurred always after hand-ball contact.

### Catching performance in relation to movement characteristics on a trial-by-trial basis

Finally, we focused on the identification of the possible sources of errors in the execution of the catching movement affecting the performance on individual trials. This information was captured by the β coefficient in TEST 3 (i.e. the variation term), which related the catching probability to the variation of the kinematic parameter with respect to its subject-specific average value. Thus, the term evaluates directly the variation of each parameter across successful and unsuccessful trials.

No differences between successful and unsuccessful trials were observed in almost all the wrist kinematics parameters evaluated. Accordingly, the within-subject term in TEST 3 was not significant in all the T-Z conditions and all the wrist parameters analyzed, with the exception of VIMP_z_ in the T_1_Z_1_ condition, and the WPs in the T_1_Z_2_ condition (p_β_ < 0.05, Table 3).

**Fig 9:**
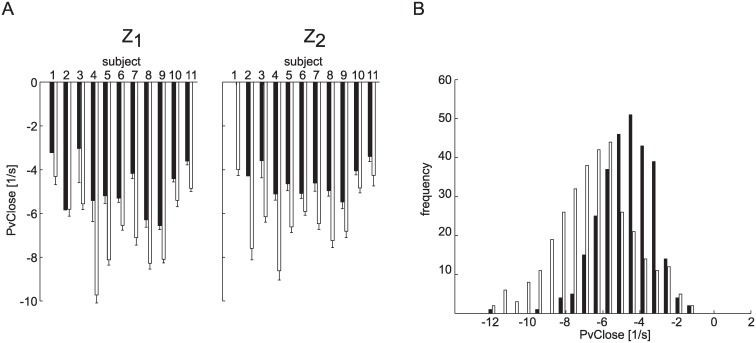
Peak hand closing velocity in grasped and non-grasped trials. (A) PvClose (mean ± SE across time flight conditions) in grasped (black bars) and touched (white bars) trials is reported separately for each participant and ball arrival height conditions (Z_1_: left; Z_2_: right). (B) PvClose frequency distribution for grasped (black bars) and touched (white bars) trials from all participants and experimental conditions. The distribution is shifted towards the left (more negative velocities, i.e. higher hand closing speed) for touched trials.

On the contrary, some of the grasping kinematic parameters predicted whether or not the participant grasped the incoming ball. Catching probability increased significantly in relation to an increase of the PvClose, peak closing velocity, that is for lower PvClose in absolute value (β > 0, p < 0.01, Table 3) in all experimental conditions. In fact, participants closed their hand faster in unsuccessful than in successful trials. Fig 9A shows PvClose (mean ± SE across flight duration conditions) for each participant and ball arrival height conditions, separately for successful (black bars) and unsuccessful trials (white bars). A similar distribution was observed also for the PaClose parameter, which occurred before impact for all participants and experimental conditions (not shown).

**Fig 10:**
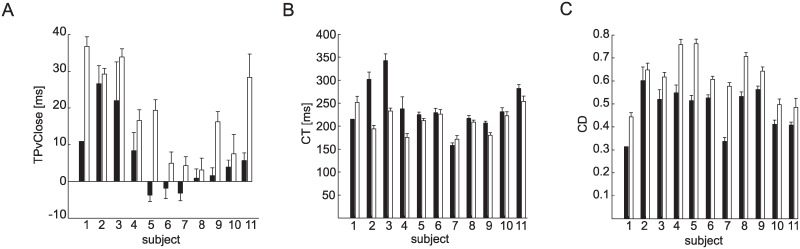
Average grasping parameters across all trials for each participants and catching outcomes. Mean ± SE (across experimental conditions) values over grasped (black bars) and touched (white bars) trials are reported separately for each participant. (A) TPvClose, time of the peak of the MCP closing velocity from impact. (B) CT, closing time. (C) CD, total flexion excursion.

Significantly larger timing errors in the control of the peak closing velocity were also observed when participants failed to catch the ball (Fig 10A). In particular, TPvClose was significantly larger in the unsuccessful than successful trials in the T_3_Z_1_ T_1_Z_2_ and T_3_Z_2_(β < 0, p_β_ < 0.01, Table 3).

CT, closing time, was higher for unsuccessful than for successful trials in the T_2_ conditions (β > 0, p_β_ < 0.05, Table 3). However, further analysis indicated that this result was ascribed to participants S_2_, S_3_ and S_4_, (Fig 10B). By fitting a GLMM model separately on each participant we observed that in the case of participants S_5_-S_11_, the variation term was not significant (p_β_ > 0.05). No differences between successful and unsuccessful trials were observed in the other T-Z experimental conditions (Table 3). CD was larger for unsuccessful than successful trials (β < 0, p_β_ < 0.001 in all the experimental conditions). Not surprisingly, the hand closed more when participant did not catch the ball (Fig 10C).

No differences across the two score groups were observed for the other grasping parameters analyzed (Table 3).

## Discussion

By examining grasping and wrist kinematics of eleven participants during a one-handed catching task, we have identified specific factors affecting catching performance. While all participants were able to intercept the ball trajectory (i.e. to touch the ball) in over 90% of cases, they differed greatly in their ability of grasping the ball as they showed a success rate ranging from 2% up to 85%. We found that it was possible to relate the outcome of the interceptive action to the variation of specific kinematic features both on a trial-by-trial basis and depending on their subject-specific average value. A higher hand closing speed distinguished between touched and grasped trials. A proper triggering of the enclosing phase of the grasping movement, an accurate alignment of the peak of the hand closing speed to the impact event, and a lower wrist vertical impact velocity characterized the performance of good catchers with respect to that of poor catchers. These factors entail different aspects of the planning and control of grasping and they are related to either the spatial or the temporal coordination of the interceptive action.

### Grasping movement characteristics

In contrast to the hand transportation component, showing different strategies for positioning the hand in the "catch zone", a stereotyped strategy was observed for the grasping component in our task. Hand closure was analyzed in terms of flexion/extension of the index MCP joint. As the fingers closed around the ball the MCP joint flexed passing from its maximum extension angle to its maximum flexion angle. All participants modulated the grasping kinematic parameters according to ball flight conditions in a similar fashion. Inter-individual variability was also present in all the parameters evaluated, but we observed a stable behavior within each participant. The initiation of the grasping movement changed depending on the ball arrival height but no significant variation with ball flight duration was observed (Table 2). These results are in accordance with many previous studies on prehension in catching [1, 3–5, 7, 9, 10, 35–37].

An open question in the study of visuomotor coordination is whether the control of interceptive actions relies on model-based predictive processes or on continuous on-line visual guidance [38, 39]. The latter hypothesis postulates that the control of the grasping behavior in a one-handed catching involves the perceptual measure of the remaining time-to-contact [35, 37]. Above all the possibilities (see [40] for a review) the inverse of the rate of expansion, τ [41], has been considered the primary source of information used to trigger the hand closure initiation [35]. However, τ-based models of grasping control introduced so far are controversial since their accuracy depended on whether the participants kept the grasping hand stationary or not during catching [35, 37]. According to the model-based approach participants use a combination of visual information and a priori knowledge of ball motion to predict its arrival characteristics and to generate an anticipatory response. In line with this second view, an earlier analysis on eye movements with data from seven of the eleven participants presented in the present analysis (respectively S_1_, S_3_, S_4_, S_5_, S_6_, S_8_, S_9_, S_10_) showed that participants stop tracking the ball in the final part of the flight, i.e. ~120 ms prior to impact [27], which coincides with the time interval from hand closure initiation to hand/ball contact observed in our data (i.e. the TOnClose parameter in Fig 5). These results suggest that prior to grasping initiation participants selected a specific motor plan to bring the fingers from maximal hand aperture to the final configuration at impact, and that no visual feedback was used to adjust the on-going hand closing.

Given the ballistic nature of the movement, the grasping motor plan was likely identified by selecting the movement time duration and finger flexion amplitude [12]. Infinite combinations were possible at this stage, as participant could choose to impact the ball both during the grasping movement acceleration or deceleration phases. Our data show that participants timed the peak of the hand closing velocity always in correspondence of the impact event (Fig 4). We wondered whether this temporal correspondence could have been related to the physics of the hand-ball interaction. At contact, the ball could have impacted the hand with such a high force to determine a sudden deceleration of the index joint movement. However this is unlikely considering the ball speed at impact (range [4.97, 11.96] m/s) and the much smaller ball mass with respect to the hand mass (~17 times smaller, ball mass = 20 gr, hand mass = 350 gr, [42]). Moreover, we observed a similar temporal correspondence in missed trials in which no contact between the hand and the ball physically occurred (Fig 4, green lines). Interestingly, a similar behavior has been reported in other studies characterizing rapid interceptive actions that did not involve ball capture, such as punching [43–45] or hitting a target with a tool [46–48]. In line with the present results, participants intercepted the target in correspondence with the time of peak velocity of the end-effector. Thus, the same strategy applied to different end-effectors, i.e. the hand [43–45], the hitter tool [46–48] or the fingers in our case, depending on the task requirements. A possible explanation of our results is that moving faster allowed participants to reduce the effects of timing errors [12, 13, 46, 49, 50], and thus it is likely that contacting the ball at the time of highest hand closing speed increased success probability.

### Sources of grasping movement errors affecting catching performance

The fact that the ball was touched in at least 90% of the cases (Fig 3), indicates that the fine tuning of the grasping was the critical aspect affecting success in our interceptive task. The ability of capturing the ball was related to different aspects of grasping movement execution. In particular, some features distinguished a poor catcher from a good catcher (TOnCLose and TPvClose parameters) and other features predicted the catching outcome on a trial-by trial basis, i.e. whether the participant grasped or touched the ball (TPvClose and PvClose parameters). Poor catchers were inaccurate at triggering the enclosing phase of the grasping movement and at timing the peak of the hand closing velocity in correspondence of the impact event. These results suggest that the main source of errors in the case of our worst catchers was of temporal nature and they might reflect both inaccuracy in ball motion perception, causing inaccurate prediction of the remaining time to contact, and in the ability at generating movements of pre-programmed duration and amplitude. Several studies indicated that the level of expertise in different ball disciplines is strongly related to a more accurate prediction of the ball motion, to a higher sensitivity to the many sensory inputs involved in the estimation process, and to the ability at paying attention to salient cues of the ball motion [20–23, 51]. All these factors may compensate for the delays involved in neural processing of visual motion and may help to reduce the reaction and execution time in the case of rapid interceptive actions. Similar considerations might apply to the participants involved in the present experiment, although an evaluation of their level of expertise in ball disciplines was out of the scope of the study.

**Fig 11:**
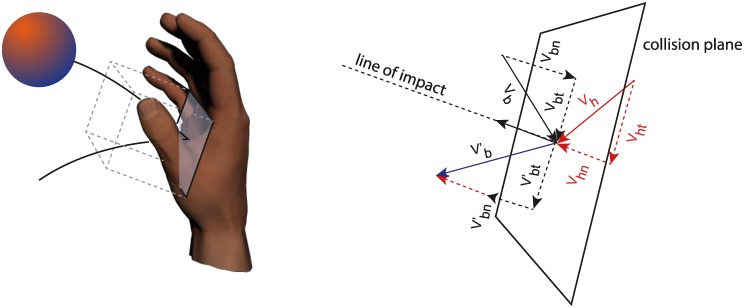
Geometry model of the hand-ball collision. Left: schematic illustration of the catchable volume. The approaching ball could be captured by participants when it was within a specific volume (here represented by a parallelepiped) relative to the hand palm. The dimensions of this volume depend on the fingers length and the size of the inner surfaces of the hand. The approaching ball could be captured at the boundary of the catchable volume with a grasping movement in which the hand-ball contact occurs along the fingertip surface. Alternatively, contact could occur along the entire finger surface when the ball was further inside the hand. Right: schematic illustration of the physics of the hand ball collision (see text).

Differences between grasped and touched trials were mainly ascribed to errors in the modulation of both the TPvClose and the PvClose parameters. Thus, when participants failed to capture the ball it was because they delayed the moment of the peak speed with respect to impact and because they closed their hand too fast. The first type of errors was related to the temporal aspects in the grasping movement discussed above, which were likely reflecting inconsistencies in the participant's ball motion prediction and noise in the sensory integration. The second type of errors could be a direct consequence of the physical interaction with the soft ball at impact, which determined a slowing of the hand motion in the case of grasped trials. However, the peak closing acceleration (PaClose), which occurred always before hand–ball contact, was larger in the case of touched trials than in grasped trials (β > 0, Table 3), thus showing a relation with catching performance similar to that of the peak closing velocity (PvClose). It is therefore more likely that the larger peak speed values in the case of touched trials were related to a misprogramming of the required hand closing distance (CD). In fact, the duration of the hand closure (CT) was a stable movement feature in our participants, as in most conditions it was not directly related to the catching outcome (i.e. β coefficient in TEST 3 was not significant in 4 out of 6 conditions). Given the ballistic nature of the grasping movement discussed above, we suggest that the large PvClose values observed in the case of the touched trials were the effect of an overestimation of the required closing distance (CD) rather than of the movement duration (CT). Moreover, because the participants intercepted the ball in correspondence with the time of peak speed, that is, almost midway between the maximal hand aperture and the final hand closing configuration, a misprogramming of the closing distance was highly related to an error in the hand closure configuration at impact. Participants failed to grasp the ball because the hand aperture was so tight at contact to impede the ball passage for the final gripping phase. This type of error might reflect an imprecise evaluation and execution of the necessary hand closing excursion due to sensory-motor noise which contributes to movement variability [52–54]. Yet, one would expect to also see errors associated with a movement amplitude underestimation, consistent with the presence of lower PvClose values in touched than in grasped trials, and thus a bimodal distribution of PvClose values for touched trials. A possible explanation for the observed unimodal distribution of PvClose for the touched trials (Fig 9, panel B), is that, in case of underestimated PvClose, participants were able to capture the ball also after it impacted on the palm and bounced off, hence gaining extra time for grasping motion execution. Similar to other interceptive tasks [12, 13, 47], ball capture in our experiment required to execute hand closing within a limited time window, which depended on ball velocity, size of the ball, the size of the hand defining a "catchable volume" (Fig 11). For a successful grasping movement, a spatial error could be tolerated as long as the ball was within this catchable volume when the participants reached the final grip configuration. When CD, closing distance, was overestimated, the ball did not penetrate perfectly inside the hand at impact. In this case, the admissible spatial error was related to the time taken by the ball to go through the catchable volume. In contrast, when CD was underestimated the fingers at contact did not adequately grip the ball. However, because the ball bounced off the palm, there was an additional time to complete the hand closure, specifically the time it took the ball to cover the catchable volume after the bounce.

### Some wrist movement strategies may facilitate grasping

We compared wrist kinematics of participants presenting different catching performances and we observed that some movement strategies were more effective than others. Participants with a low positive or a negative vertical wrist velocity values at impact caught a larger number of balls than those with high positive values (Fig 7). However, all participants arrived with the hand "at the right time and place" in almost the 90% of the cases. We suggest that some hand transportation strategies facilitated grasping more than others. One possibility is that a lower or negative hand vertical velocities at impact reduced the post-bounce ball velocity and thus increased the time window available to close the fingers. We consider the hand-ball collision problem of our task similar to that of a ball impacting obliquely on a wall moving at **v**_h_ velocity, which is reasonable given the large differences between the two masses. According to the physics of inelastic collision, assuming no friction at contact, the impulsive force generated at impact acts along the normal to the collision plane represented by the palm (line of impact, see Fig 11) and no external forces are applied along the plane of impact. The ball velocity component along the plane remains unchanged after the bounce (*v*_*bt*_ = *v*′_*bt*_). In contrast, the velocity component along the line of impact changes according to: *v*_*b*′*n*_ = −*CR*·*v*_*bn*_ +(1+*CR*)·*v*_*hn*_ where CR is the coefficient of restitution that takes into account the elastic properties of the collision between the ball and the hand. It follows that when the ball and the hand velocities are concordant along the line of impact, the post-bounce ball velocity *v*′_*bn*_, thus *v*′_*b*_, is lower than the pre-bounce ball velocity *v*_*bn*_, thus *v*_*b*_. Moreover, the larger *v*_*hn*_ the lower the *v*′_*bn*_. When instead *v*_*bn*_ and *v*_*hn*_ are discordant, the post-bounce ball velocity in the direction of the line of impact is larger than the pre-bounce ball velocity, and in particular the larger the *v*_*hn*_, the larger the *v*′_*bn*_. In other words, grasping probability in our task is likely maximized exploiting a "buying time" strategy which relies on the control of the hand velocity at impact. In this context, arriving on the ball from above as in the *hook-like* strategy observed in most of our best catchers (i.e. S_7_, S_9_, S_10_, S_11_, S_12_), slowed down the ball in the post-bounce trajectory because the descending ball and the hand had both negative vertical velocity components at impact. Similarly, another way to reduce the post-bounce velocity of the ball and to increase the period available to complete the grasping was to stop the hand at impact (i.e. the *stop-on-the-ball* strategy) or to move the hand backwards and with lower vertical velocity as in the case of high ball arrival height conditions. In contrast, when participants hit the ball from below and with a large positive vertical velocity (i.e. *hitting-like* strategy) the ball post-bounce velocity was larger than the pre-bounce velocity, and thus the time to complete the ball capture was reduced with respect to the other cases. Indeed, this behavior was often observed in our worst catchers (S_1_-S_4_, see Figs 6 and 7).

Although based on an approximate model of the physics underlying the hand-ball interaction in our task, this interpretation indicates that the wrist movement strategies observed in our best catchers might have facilitated task performance. Larger spatial error in the grasping movement plan were likely tolerated because of the larger available time window. However further dedicated experiments are required to draw more definitive conclusions on the interplay between hand transportation and grasping control strategies, as well as their dependence on the level of catching ability. For instance the hand palm orientation and the choice of the impact point along the ball trajectory, which we showed to be different across participants and conditions [6], should also be taken into account. Moreover, possible small differences in the location of the hand at impact, which caused fluctuation of the ball contact point on the hand palm and fingers surfaces, should be characterized more systematically. In this context it would be also interesting to assess whether and how the observed variability across participants in our experiment are related to individual differences in the acquisition of similar novel tasks [55].

## Conclusions

In summary, we showed that fine-tuning of the grasping movement is the aspect of one-handed ball catching which affected performance most. In the same task, we previously showed that participants stopped tracking the ball in the final part of its flight [27], i.e. when the grasping movement occurred. Thus, it is likely that participants took advantage of target motion information for online adjustment of the wrist movement while they used a pre-programmed grasping response triggered at a fixed time before impact. Both movement time and excursion of such grasping response were likely pre-programmed. Different types of errors were related to the individual skill level. Poor catchers among our participants mainly failed in correctly timing the grasping movement. In particular, they delayed grasping onset in the case of fast balls and they were inaccurate at aligning the time of the peak closing speed with the time of impact. These results are consistent with a less accurate estimation of the remaining time-to-contact used to initiate the movement, and with an imprecise generation of the appropriate motor patterns from the required commands. Unsuccessful performances in the case of our best catchers, instead, were mainly characterized by spatial errors in the generation of the appropriate hand closing amplitude which might reflect the effects of sensory and proprioceptive noise. In manual interception the temporal and spatial representation of the movement are separate and explicitly represented in the control of the action [56]. Nevertheless a spatial error can also be represented as a temporal error, and vice versa [12]. Thus, participants could take advantage of their proximal joints kinematics to gain more time for the execution of the grasping movement, and hence tolerate larger amplitude errors. We provided evidence for a similar mechanisms in the case of our best catchers. However, future investigations are required to corroborate this hypothesis.

## Supporting Information

S1 Appendix The random-effects structure in the Generalized Linear Mixed Models. (DOCX)

S1 Fig The average values of the TOnClose parameter (mean ±SE, across trials) in the different T and Z experimental conditions. Data are reported separately for each individual.
(EPS)

S1 File Results of Repeated Measure ANOVA. (DOCX)
