# Supplementary material for: Grasping in One-Handed Catching in Relation to Performance
Source: PLoS One. 2016 Jul 8;11(7):e0158606. doi: 10.1371/journal.pone.0158606 (PMC4938428; doi:10.1371/journal.pone.0158606)
Supplement: S1 Appendix — (DOCX) [file pone.0158606.s001.docx]

**The random-effects structure in the Generalized Linear Mixed Models**

GLMM are often used to fit clustered dataset which include repeated measure from several subjects. GLMM can be thought as a series of interrelated regressions aimed at modeling sources of variability at multiple levels of analysis such as the experimental stimuli and the subject level.

A GLMM-based test consists in the estimation of a baseline regression, underlying the behavior of the population and being characterized by grand-average intercept and grand average slope terms (i.e. fixed effects), and in the simultaneous estimation of subject-specific changes with respect to the baseline level (i.e. random effects). This can be achieved including by-subject random intercept and by-subjects random slopes terms. To give an example, with reference to the present analysis, we can consider the following model that fits a generic motor response, i.e. ν, in TEST 2:

$\nu_{\text{ij}}=\text{β}_{0}+\text{(β}_{\text{T}}+\text{S}_{\text{Ti}}\text{)t}_{\text{j}}+\text{(β}_{\text{Z}}+\text{S}_{\text{Zi}}\text{)z}_{\text{j}}+\text{S}_{\text{0i}}+\text{ε}_{\text{ij}}$ ( S2.1)

where the β_0_ is grand-average intercept, and the β_T ,_ β_Z_ coefficients are the grand-average slope terms of the regression. By including the S_0i_ term, the linear predictor of each subject is shifted from the baseline level β_0_. Similarly, the S_Ti_ and S_Zi_ terms capture how much subject's effect deviates from the population behavior (i.e. β_T ,_ β_Z_). S_0i,_  S_Ti_, and S_Zi_ terms define the source of clustering of our data and hence they are representative of the *random effect structure* of model, which may be different depending on the specific response variable under analysis (see Table 2 in the main text). Note that it is possible to compute the new coefficients of the regressions for each subject, but no statistical tests are available for direct comparisons in the manner of post-hoc test as in the ANOVA. The contribution of fixed and random effects on the total variance explained by the model can be evaluated by calculating both the marginal (i.e. the proportion of variance explained by the fixed factor) and the conditional (i.e. the proportion of variance explained by both the fixed and random factors) R^2^ parameters according to the following formulas:

$R^{2}_{m\arg inal}= \frac{\sigma_{fix}^{2}}{\sigma_{tot}^{2}}$ (S2.2)

$R^{2}_{conditional}= \frac{\sigma_{fix}^{2}+\sigma_{rand}^{2}}{\sigma_{tot}^{2}}$ (S2.3)
