# Supplementary material for: Grasping in One-Handed Catching in Relation to Performance
Source: PLoS One. 2016 Jul 8;11(7):e0158606. doi: 10.1371/journal.pone.0158606 (PMC4938428; doi:10.1371/journal.pone.0158606)
Supplement: S1 File — (DOCX) [file pone.0158606.s003.docx]

**S1. File. Results of Repeated Measure ANOVA**

Repeated-measures ANOVA (RM-ANOVA with three levels for T, ball flight duration, and two levels for Z, arrival height, as within-subjects factors) was used to test for statistically significant changes of each movement features evaluated as a function of the ball flight duration and arrival height (*aov* function in R package, version 1.0–5 http://RAN.R-project.org/package), and the results are reported in the Table A.

**Table A Results of the RM-ANOVA test. Statistical main effects of T and Z factors are reported separately for every dependent variable.**

| ANVOAs | **T** | **Z** |
| --- | --- | --- |
| Grasping parameters |  |  |
| TOnClose | ** | 0.05 |
| CD | ** | ** |
| TPvClose | 0.11 | *** |
| PvClose | ** | * |
| TPaClose | 0.08 | *** |
| PaClose | ** | * |
| CT | 0.16 | 0.66 |
| Wrist parameters |  |  |
| LT | 0.07 | 0.98 |
| WPs | *** | *** |
| TWPs | *** | 0.75 |
| VIMP_x_ | 0.6 | *** |
| VIMP_z_ | *** | *** |

***: p_value <0.05; **: p_value<0.01; ***: p_value<0.001.**

RM-ANOVA and GLMM are both linear models and thus, not surprisingly, they produced comparable results. In fact, similar main effects were observed for the T and Z factors with both analyses (see Table 2 and Table A in S1 File). However, a few discrepancies as for the TOnClose and TPaClose parameters were also observed. We believe this occurred for several reasons. First, different algorithms were used to fit data. Specifically, ANOVA is based on the least-square approach, whereas the GLMMs is based on the maximum likelihood policy (Barr DJ et al. 2013). Second, the two methods make different assumptions on the error term distributions. RM-ANOVA assumes that the random error terms are normal, independent, and each with constant variance. By contrast, the GLMM models consider that the random error terms are normal, possibly correlated, and with possibly unequal variances. Third, the outcomes of the ANOVA are not reliable in the presence of multiple missing data points, as in the case of TEST 2 where we compared subjects with different numbers of successful trials. Finally, considering by-subjects adjustments of both the slope and the intercept coefficients of the regression, GLMM accounted for a more complex random effect variance than RM-ANOVA. In this respect, S1 Fig. reports the average TOnClose values (mean ± SE across trials) in the T and Z conditions separately for each participant. Some participants showed a trend in the distribution of the TOnClose parameter across the T conditions (S_3_, S_5,_ S_8_, S_11_). However, this trend was not clearly observed in the other participants. Thus, the relation between the TOnClose variable and the T factor changed depending on the subject. This information was captured by the GLMM in TEST 2, for which the random slope effect for the T factor was found to be significant in the case of TOnClose parameter (Table 2), but it could not be captured with a RM-ANOVA analysis.
